# Green Engineering of Bio-Epoxy Resin: Functionalized Iron-Oxide Nanoparticles for Enhanced Thermal, Mechanical, Surface and Magnetic Properties

**DOI:** 10.3390/polym17131819

**Published:** 2025-06-29

**Authors:** Klementina Pušnik Črešnar, Julio Vidal

**Affiliations:** 1 Faculty of Mechanical Engineering, University of Maribor, 2000 Maribor, Slovenia; 2 Faculty of Chemistry and Chemical Engineering, University of Maribor, 2000 Maribor, Slovenia; 3 Faculty of Polymer Technology, 2380 Slovenj Gradec, Slovenia; 4 Fundación Aitiip, Pol. Ind. Empresarium C/Romero, 12, 50720 Zaragoza, Spain; julio.vidal@aitiip.com

**Keywords:** bio-based epoxy (nano)composites, curing behavior, dynamic mechanical properties, surface properties

## Abstract

In the pursuit of environmental sustainability, reduced emissions, and alignment with circular economy principles, bio-epoxy resin nanocomposites have emerged as a promising alternative to traditional petroleum-based resins. This study investigates the development of novel bio-epoxy nanocomposites incorporating iron-oxide nanoparticles (Fe_2_O_3_, MnP) as multifunctional fillers at loadings of 0.5 wt.% and 3.0 wt.%. MnP nanoparticles were synthesized and subsequently functionalized with citric acid (MnP-CA) to enhance their surface properties. Comprehensive characterization of MnP and MnP-CA was performed using X-ray diffraction (XRD) to determine the crystalline structure, attenuated total reflection Fourier-transform infrared spectroscopy (ATR-FTIR), thermogravimetric analysis (TGA), and zeta potential measurements to confirm surface functionalization. The bio-epoxy resins matrix (bio-EP), optimized for compatibility with MnP and MnP-CA, was thoroughly analyzed in terms of chemical structure, thermal stability, curing behavior, dynamic–mechanical properties, and surface characteristics. Non-isothermal differential scanning calorimetry (DSC) was employed to evaluate the curing kinetics of both the neat (bio-EP) and the MnP/MnP-CA-reinforced composites, offering insights into the influence of nanoparticle functionalization on the resin system. Surface zeta potential measurements further elucidated the effect of filler content on the surface charge and hydrophilicity. Magnetic characterization revealed superparamagnetic behavior in all MnP- and MnP-CA-reinforced (bio-EP) composites. This research provides a foundational framework for the design of green bio-epoxy nanocomposites, demonstrating their potential as environmentally friendly materials and representing an emerging class of sustainable alternatives. The results underscore the viability of bio-epoxy systems as a transformative solution for advancing sustainable resin technologies across eco-conscious industries.

## 1. Introduction

Epoxy resins (EP) are among the most widely used thermoset polymers in the polymer composite industry. The main structure of EP includes epoxide terminal groups, which form a highly crosslinked network. The term “epoxy” broadly refers to both the basic and cured forms of EP) [1,2,3,4]. Since 1935, when Sylvan Greenlee and Pierre Castan first synthesized conventional EP based on bisphenol A, EP usage in industries has shown a growing trend. The global EP thermoset market was valued at USD 7.0 billion in 2015 and reached USD 10.2 billion by 2021. According to SNS Insider Research, the EP market was valued at USD 24.1 billion in 2022 and is projected to increase to USD 42.28 billion by 2030 [5]. To date, the most used EP is synthesized by reacting epichlorohydrin with bisphenol A, resulting in Diglycidyl Ether of Bisphenol A (DGEBA). In general, a specialized class of highly reactive pre-polymers, or polymers featuring epoxide groups in their molecular structure, is known as EP, epoxides (in Europe), α-epoxy, or 1,2-epoxy. Epoxies can self-polymerize via catalytic homo-polymerization to form crosslinked macromolecular structures, or they can react with various co-reactants, such as polyfunctional aliphatic and aromatic amines, acid derivatives (like acid chlorides, amides, esters, and anhydrides), and aliphatic and aromatic alcohols and thiols (mercaptans) [6]. Hardeners, also known as curatives or co-reactants, facilitate a crosslinking process commonly referred to as curing. When DGEBA is combined with a polyamine hardener, an exothermic addition reaction produces a fully cured, crosslinked 3D network. This curing process results in a high degree of crosslinking, which not only increases the brittleness of the cured EP but also enhances the material’s mechanical strength (due to its high tensile strength and Young’s modulus), as well as its thermal and chemical stability. In summary, although the extensive use of EP is well-recognized in applications such as surface coatings, electrical insulators, marine systems, aerospace parts, fiber-reinforced plastics, paintbrushes, electronic components/LEDs, and adhesives, their inherent brittleness and limited fracture toughness restrict their industrial applications [6,7,8,9,10]. By modifying the chemical structure of EP or adjusting curing kinetics, it is possible to vary their mechanical properties and enable them to fit into the final requirements demanded by each application. Furthermore, the addition of reinforcements and fillers such as nanoparticles in EP can transform the properties of EP through the formation of epoxy-based composites and specifically in the case of nanomaterials in nanocomposites (EP-nC), resulting in improved mechanical, optical, anticorrosive, electrical, and magnetic properties [7,9].

While conventional EP resins (nano)composites offer numerous benefits, limitations in petrochemical resources, alongside environmental and economic concerns, restrict their usage. Reducing emissions by limiting the use of petrochemical-based polymers is a crucial step toward achieving future-oriented Green Deal strategies to put us on track to climate neutrality by 2050, building a healthier and safer future. These strategies aim to accelerate the adoption of renewable raw materials, renewable energy, and sustainable production technologies to enhance the eco-friendly production of polymer materials, including both thermoset and thermoplastic plastics. Consequently, the development of new bio-based polymers, which are environmentally compatible and utilize renewable raw materials, is essential to addressing environmental concerns [2,9,11]. As a result, numerous research groups and manufacturers have dedicated significant efforts to developing bio-based epoxy resins (bio-EP) for use as matrix materials in composite structures, especially nanocomposites. In this context, the number of published studies on EP, bio-EP, EP-nC, and bio-epoxy nanocomposites (bio-EP-nC) increased to 58,866 between 2013 and September 2023, although only 116 of these studies focused on bio-EP-nC [3,5]. To achieve high-performance EP-nC—whether petrochemical-based or bio-based—careful attention is needed regarding the quality of nanofiller dispersion within the polymer matrix and the interfacial interactions between the fillers and the EP, which enhances the curing process. Due to the tendency of nanoparticles to agglomerate and their large specific surface area, achieving good interfacial compatibility and interactions between the EP matrix and nanofillers remains challenging [9]. Poor interfacial compatibility can create points that initiate cracks, making the dispersion and interaction of nanoscale fillers crucial factors in their effective application. With their high surface area-to-volume ratio, surface functionalization of nanoparticle fillers can effectively improve interfacial interaction between the filler and the EP polymer. Surface treatment of nanofillers is thus essential to resolve dispersion issues and enhance EP-filler interactions, thereby improving the final properties of the composite. Typically, nanoparticle surface properties are modified to suit specific application needs. This is often performed by intermolecular forces or adsorbing organic molecules onto the nanoparticle surfaces to tailor features such as surface charge, hydrophilicity or hydrophobicity, and the presence of specific functional groups. Such modifications not only improve compatibility between nanoparticles and EP matrices but also allow for precise control over particle dispersion, stability, and reactivity within polymer environments and causes enhanced curing behavior [12]. It is generally reported about the role of surface modification of nanoparticles that lies on interplay between the state of cure and the ultimate properties of EP-nanocomposites. Despite that curing EP depends on curing time, temperature, type of hardener, the type, shape and surface chemistry of nanoparticles have an important role on curing behavior of epoxy nanocomposites due to nanoscale dispersion of filler. The dependence on the incorporation of either micro-or nanofillers that cause changes on EP composites curing behavior has been extensively studied in numerous investigations. Surface functionalization of nanoparticles with starch and cyclodextrin provides enhanced curing process due to through retardation of gelation and vitrification in the diffusion-controlled region above a critical conversion [13,14,15,16,17]. By adjusting these properties, nanoparticles can be tailored for improved performance in EP-nC applications, including drug delivery, catalysis, electronic devices, coatings, and composite materials. Customizing surface characteristics in this way allows for more effective integration into bio-EP systems, curing process, maximizing functionality and efficiency across various technological and industrial applications. In summary, the literature highlights the great potential of nanoparticles in enhancing the properties and performance of bio-EP polymers [18].

However, although many studies have explored the incorporation of nanofillers into petrochemical-based EP resins—such as nanosilica, nanoclay, carbon-based nanomaterials, and more recently, metallic nanofillers like iron oxide, copper, zinc, and aluminum oxides [11,18,19,20,21,22]—our research is the first to emphasize the significant role of iron-oxide nanoparticles with citric acid as a natural coupling agent in bio-EP. The future of bio-EP appears promising, driven by innovations that prioritize sustainability without compromising performance. Yet, addressing challenges related to performance limitations (due to enhanced thermal and mechanical properties), variability in raw materials (requiring quality control and standardization processes to ensure uniform properties), processing complexities (as the synthesis and curing processes for bio-EP can be more intricate than those for conventional resins), economic viability, and recycling and end-of-life considerations will be crucial for advancing bio-EP resin production. Continued research to improve material properties, standardize production processes, enhance economic viability, and develop recycling methods will be essential for making bio-EP a mainstream alternative across various applications.

Iron-oxide nanoparticles, specifically maghemite (γ-Fe_2_O_3_) (MnP), are considered safe and have been approved by the U.S. Food and Drug Administration (FDA) for in vivo applications. Due to their biocompatibility, environmental friendliness, and relatively high saturation magnetization (60–80 emu/g at room temperature), MnP nanoparticles have demonstrated valuable applications, including magnetic data storage, catalysis, targeted drug delivery, and electrochemical activity [23,24,25]. Whether unmodified or functionalized with citric acid (CA)—a naturally unique compound—these nanoparticles can act as coupling agents, enhancing nanofiller dispersion, interfacial interaction, and providing improved curing mechanical properties and superparamagnetic behavior in bio-EP. This novel approach has not been previously reported. The development of bio-EP composites filled with MnP or MnP-CA could expand their application range to include microwave absorption [26,27], magnetic resonance imaging (MRI) [28], electromagnetic interference shielding, flexible electronics, magneto-optical storage [29], biomedical sensors [30,31], catalysis, magnetochromatic materials [32], magnetic shape memory [33], and heavy metal removal [34]. The literature review reveals that several studies have examined the use of iron-oxide nanoparticles (such as maghemite and magnetite) as fillers in EP-polymer composites. Incorporating Fe_3_O_4_ or Fe_2_O_3_ nanoparticles with varying filler loadings and surface treatments—including (3-Aminopropyl) triethoxysilane (APTES), polydopamine (PDA), silane, polypyrrole (PPy), chitosan and functionalized carbon or oxide coatings—has been shown to enhance nanoparticle dispersion, curing kinetic, increase tensile strength, and improve fracture toughness in EP-nC nanocomposites [9,35,36,37,38]. These treatments also influence the curing process and the magnetic properties (e.g., saturation magnetization, Ms) of the Fe_3_O_4_-based EP composites. It was accepted that the curing of EP–magnetite–chitosan nanocomposites was driven by the catalytic effect contributed from hydroxyl groups [38]. While many studies focus on iron-oxide nanoparticle EP composites, no work has been reported on iron-oxide bio-EP nanocomposites. Furthermore, no systematic studies have evaluated the effects of using bare or naturally surface-treated MnP, MnP-CA, such as those treated with CA, on the cure kinetics, dynamic mechanical, thermal, and surface properties of bio-EP composite materials. Non-isothermal modelling measured with differential scanning calorimetry (DSC) has also not been applied to investigate the cure kinetics process of bio-EP composites in the presence of MnP and MnP-CA.

## 2. Materials and Methods

Thus, this investigation provides an in-depth evaluation of the potential use of MnP and MnP-CA (with varying weight percentages) as natural nanoparticle fillers for bio-EP nanocomposites. These findings not only pave the way for future research on bio-EP composites derived from natural by-products but also establish a foundation for industrial applications of this sustainable approach, advancing the broader goal of an eco-friendlier thermoset plastics industry. In this study, iron-oxide bio-EP nanocomposites reinforced with MnP and MnP-CA nanoparticles were synthesized. The MnP nanoparticles were produced via a coprecipitation method and surface-functionalized with CA. Systematic investigations were conducted to examine the effects of nanoparticle loading and surface functionality on the bio-EP resin’s storage/loss modulus and tan delta. Mechanical properties, including dynamic mechanical analysis (DMA), were evaluated, and thermogravimetric analysis (TGA) was performed to assess the thermal stability of the bio-EP-MnP and bio-EP-MnP-CA composites. For comparison, samples containing as-received MnP were also prepared. To further characterize the curing reaction in bio-EP-MnP and bio-EP-MnP-CA composites, differential scanning calorimetry (DSC) was used to monitor the thermoset curing process, with cure kinetics evaluated by non-isothermal DSC methods at different heating rates. Additionally, the effects of incorporating MnP and MnP-CA into bio-EP formulations were systematically studied through comprehensive analyses of the Attenuated total reflectance Fourier transform infrared spectroscopy (ATR-FTIR), DSC, and TGA results as well. Surface zeta potential was carried out to characterize the surface properties of the bio-EP-MnP and bio-EP-MnP-CA composites.

### 2.1. Materials

The commercial bio-EP used in this study as a thermoset matrix reinforced with iron-oxide nanoparticles was Infugreen 810, paired with the 4770 hardener. Both components were purchased from MEL Composites (Barcelona, Spain). Infugreen 810 is a partially bio-based epoxy resin system, developed by Sicomin (Chânteauneuf-les-Martigues, France) and marketed under the GreenPoxy brand, featuring a bio-based carbon content of 29%. The bio-EP component alone contains up to 38% plant-based carbon. The Infugreen resin system is a bicomponent system, requiring 100 g of Infugreen 810 resin and 29 g of the 4770 hardener. All consumables used in the study were also provided by MEL Composites (Barcelona, Spain).

The chemicals used for synthesizing the iron-oxide nanoparticles and for modifying the nanoparticles with CA included iron (III) sulfate hydrate (97%, reagent grade), iron (II) sulfate heptahydrate (ACS (Washington, DC, USA), 99%), ammonium hydroxide (NH_4_OH) (aq) (25%), sodium hydroxide (98%), and citric acid, all of which were purchased from Sigma-Aldrich (Amsterdam, The Netherlands).

### 2.2. Methods

#### 2.2.1. Synthesis of Iron-Oxide Nanoparticles and Modified Iron-Oxide Nanoparticles with Citric Acid

In general, nanoparticle synthesis was based on coprecipitation methods. The iron-oxide nanoparticles (MnP) were synthesized as described in our previous work [39] via the coprecipitation of Fe^3+^ and Fe^2+^ ions from an aqueous solution using aqueous ammonia. Specifically, MnP nanoparticles were precipitated from an aqueous solution of FeSO_4_ (0.027 mol/L) and Fe_2_(SO_4_)_3_ (0.0115 mol/L) by adding a concentrated ammonia solution (25%). The pH of the solution was adjusted to 11.6 through the addition of ammonia under vigorous mixing. After an aging period of 30 min, the MnP particles were magnetically separated and washed multiple times with a diluted ammonia solution at a high pH of 10.5. This high pH provided a strong negative surface charge on the nanoparticles (which focuses on the zeta-potential variation with pH), helping to prevent strong agglomeration during washing. The MnP modification was achieved by treatment with CA under atmospheric conditions, as reported in Reference [40].

#### 2.2.2. Synthesis of Bio-Epoxy Composites Cured with Unmodified and Modified Iron-Oxide Nanoparticles

The neat bio-epoxy resin (bio-EP) was prepared using a commercial formulation with 100 g of Infugreen epoxy resin and 29 g of hardener at an ambient temperature. The mixture was stirred for 0.5 h to obtain a homogeneous solution. The bio-EP was then cured overnight in an oven at 80 °C.

In the next step, epoxy resin (100 g) loaded with MnP and MnP-CA was prepared by manually stirring a calculated amount of MnP (0.5 and 3.0 wt%). To improve compatibility between the nanoparticles and epoxy components, the calculated amount of MnP was first mixed with the hardener and stirred for 1 h. Following this, the bio-EP was added and stirred for an additional hour at an ambient temperature. The prepared bio-EP, reinforced with MnP and MnP-CA, was then cured at 80 °C to obtain bio-EP composites reinforced with MnP (EP-MnP) and MnP-CA (EP-MnP-CA).

**Figure 1:**
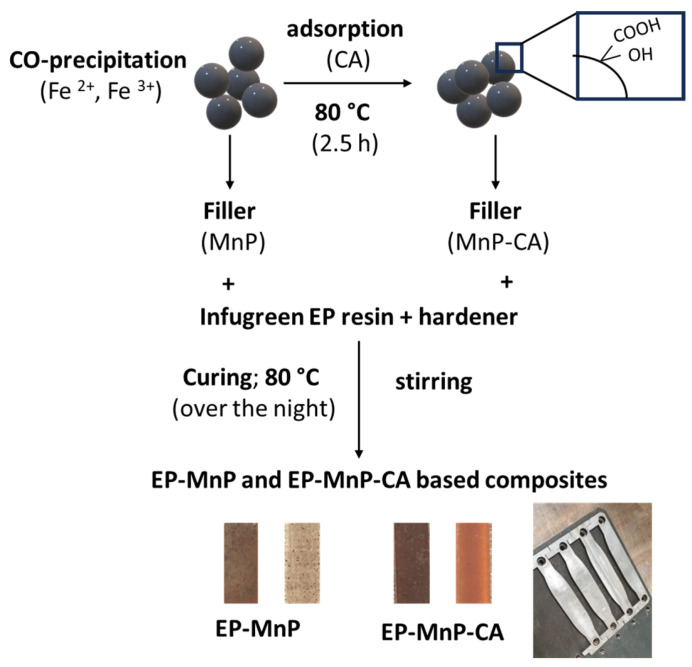
Schematic overview of the present study: the preparation of bio-EP composites filled with MnP, MnP-CA.

Finally, the neat bio-EP, EP filled with MnP, and EP filled with MnP-CA were poured into dog-bone-shaped metal molds for self-curing and shaping. After curing, all samples were removed from the molds and allowed to cool for 24 h before undergoing various characterizations. The same method was used to prepare neat bio-EP composites without any filler, which served as control samples. Detailed information about each sample can be found in Table 1 and Figure 1.

#### 2.2.3. Characterization Methods

**Characterization of nanoparticles**

The X-ray diffraction (XRD) spectra of nanoparticles (MnPs and MnP-CA) were recorded by a Siemens D5000 diffractometer using a Cu K α radiation source at 40 kV. The X-ray diffraction patterns were recorded for the angles in the range of 2θ, with a scanning step of 10/min (λ = 0.154) and measured from 20 to 60.

The mean crystallite size was determined using the widely recognized Scherrer formula:

Crystallite size [nm] = kλ/βcosθ,(1)

In this equation, λ represents the X-ray wavelength in nanometers (nm), with a typical value of 0.154 nm for Cu Kα radiation. β denotes the full width at half maximum (FWHM) of the diffraction peak, measured in radians, which is attributed to the small size of the crystallites. k is a dimensionless shape factor, commonly assumed to be 0.9.

The crystallite size was estimated by fitting the diffraction peaks with a Gaussian–Lorentzian function using OriginPro software 2018. The electrokinetic measurements of the ξ-potential of MnPs and MnP-CA aqueous suspension were performed as a function of the suspension’s pH using a Zetasizer Nano ZS (Malvern Instruments, Worcestershire, UK) equipped with a He-Ne-laser (wavelength of 633 nm) at 25 °C. The pH of the nanoparticles suspensions was adjusted with HCl (0.1 mol/L) or NaOH (0.1 mol/L).

The ATR-FTIR analysis and thermogravimetric measurements were evaluated as described in the following procedure below (in Section 2.2.4).

#### 2.2.4. Characterization of Bio-Based Epoxy Composites Cured with Nanoparticles

Attenuated Total Reflectance Fourier transform infra-red spectroscopy.

A PerkinElmer Spectrum 3 spectrometer (PerkinElmer FTIR, Omega, Ljubljana, Slovenia) was applied for monitoring the Attenuated Total Reflectance Fourier-Transform Infrared (ATR-FTIR) spectra. The spectra were recorded in the wavenumber range from 600 to 4000 cm^–1^ at room temperature (RT). Each spectrum was determined as an average of 32 scans at a resolution of 4 cm^–1^ for measuring the background. All spectra presented here were baseline corrected and smoothed after the measurement.

##### Thermogravimetric Method

Thermogravimetric analysis (TGA) of EP, EP-MnP, and EP-MnP-CA composites was carried out using a TGA/DSC 3+ (Mettler Toledo, Greifensee, Switzerland) in 40 μL aluminum crucibles. The samples of composites (6.0–0.5 mg) were measured from 25 to 800 °C in a 50 mL/min flow of N_2_ at heating rates of 10 °C/min. Continuous recordings of the sample temperature, sample mass, its first derivative, and heat flow were taken.

##### Differential Scanning Calorimetry-Kinetic Study

A METTLER TOLEDO DSC 3+ Star System (Columbus, OH, USA) was used as a determination tool of curing enthalpy of bio-EP, EP-MnP, and EP-MnP-CA composites. In addition, the study of the curing times and the conversion module of METTLER STARe System V17.00 was implemented as it allows us to predict the reaction times at different temperatures based on the software’s nth-order kinetics models and on more than 3 dynamic conversion curves by applying a constant *E*a. About 10.0 mg of samples were calibrated in standard aluminum pans. The heat temperature scan was performed with varying heating rates in the N_2_ atmosphere (20.0 mL/min) to understand the kinetics of the reactions and the enthalpy of the curing process at different heating rates (1 °C/min up to 20 °C/min).

In the DSC measurements, the degree of cure (α) ranges from 0 (completely uncured) to 1 (fully cured) and is defined as follows:

(2) $$\alpha (t)=\frac{H(t)}{H_{T}}$$

where *H*(*t*) is the enthalpy of the reaction up to time t and *H*_T_ refers to the total enthalpy of the reaction. Common DSC techniques include monitoring the heat flow over time and allowing the total reaction enthalpy to be determined by integrating the heat flow across the entire exothermic peak. In this study, non-isothermal DSC curves were analyzed for the epoxy resin system at varying heating rates, ranging from 2.0 to 20.0 K/min. As anticipated, the DSC curing process of the epoxy resin exhibited a broad exothermic peak, with the peak shifting as the heating rate increased.

##### Dynamic Mechanical Analysis

The dynamic mechanical properties (DMA) of different samples were measured using a Dynamic Mechanical Analyzer DMAQ850 from TA Instruments (New Castle, DE, USA), following ISO standards: ISO 6721-1:2019, ISO 6721-4:2019, and ISO 6721-11:2019 [41,42,43]. These measurements evaluated tensile vibration using the non-resonance method. These standards specify the procedures for determining the dynamic mechanical properties of rigid plastics within the linear viscoelastic region when subjected to oscillatory deformation on a dynamic thermomechanical analyzer (DMTA).

The ISO 6721-1:2019 standard provides a method for determining the elastic and loss moduli as a function of temperature, frequency, time, or both. A plot of the elastic and loss modulus versus temperature offers a graphical representation of elasticity and damping across temperatures or frequency variations. Depending on the experimental conditions, different ISO standards apply. For instance, ISO 6721-4:2019 covers the specific test method for tensile testing, allowing the damping properties to be measured using non-resonant forced-vibration techniques over a wide frequency range. To identify the glass transition region and determine the glass transition temperature of materials, ISO 6721-11:2019 is used.

According to these standards, a tensile vibration method was employed to determine the components of Young’s complex modulus (storage modulus, loss modulus, and tan delta or loss factor) at a specific frequency as a function of temperature. Each sample underwent two consecutive test cycles. All composites and neat bio-EP samples were tested using tensile test specimens and studied in flexure with a dual-screw film clamp. Testing was conducted at a frequency of 1.0 Hz with an oscillation strain of 0.1% and a static force of 0.01 N. The temperature range for composite measurements was 0 °C to 150.0 °C, with a heating rate of 2.0 K/min. The specimens were dog-bone-shaped, with an approximate thickness of 2.5 mm.

According to the standards, test specimens should have a rectangular cross-section with uniform thickness and width, varying no more than 3.0% along the specimen’s length. The length-to-width ratio should be sufficient to minimize the lateral constraint from the clamps, allowing for a free lateral contraction. To meet these requirements, all samples were carefully machined to the specified width and thickness. All dimensions were measured with a caliper capable of accuracy within ±1.0%, per ISO 6721-4:2019. Table 2 presents the dimensions of the samples tested. Thickness and width measurements were taken at three points along the length of each sample, with the mean value reported.

##### Zeta Potential Measurements

The surface zeta potential was evaluated by streaming potential on the SurPASS 3 instrument (Anton Paar GmbH, Graz, Austria). The Adjustable Gap Cell was used to determine the surface zeta potential of neat EP, EP-MnP, and EP-MnP-CA modified with CA, while Litesizer 500 (Anton Paar GmbH, Graz, Austria) was used for measurements of functionalized MnP. Two pairs of identical specimens, approximately 20 mm × 10 mm in size, were cut out and adhered to sample holders with double-sided adhesive tape. The distance between the opposing samples was adjusted to (110 ± 10) µm during the rising process using a 1.0 mM KCl electrolyte solution using an adjustment knob. The measurement was performed with 1.0 mM KCl, and the streaming potential was measured with the Ag/AgCl electrode. For pH adjustments during the scan measurement as a function of pH, 0.05 M HCl and KOH were used. 

##### Magnetic Properties

The response of bio-EP with MNP and MNP-CA nanoparticles was evaluated in terms of magnetic properties. The magnetizations of bio-EP-MNP/MNP-CA as a function of magnetic field strength were measured using a Lake Shore 7307 VSM vibrating sample magnetometer.

## 3. Results and Discussion

### 3.1. Characterization of Iron-Oxide Nanoparticles and Modified Iron-Oxide Nanoparticles with Citric Acid

**Figure 2:**
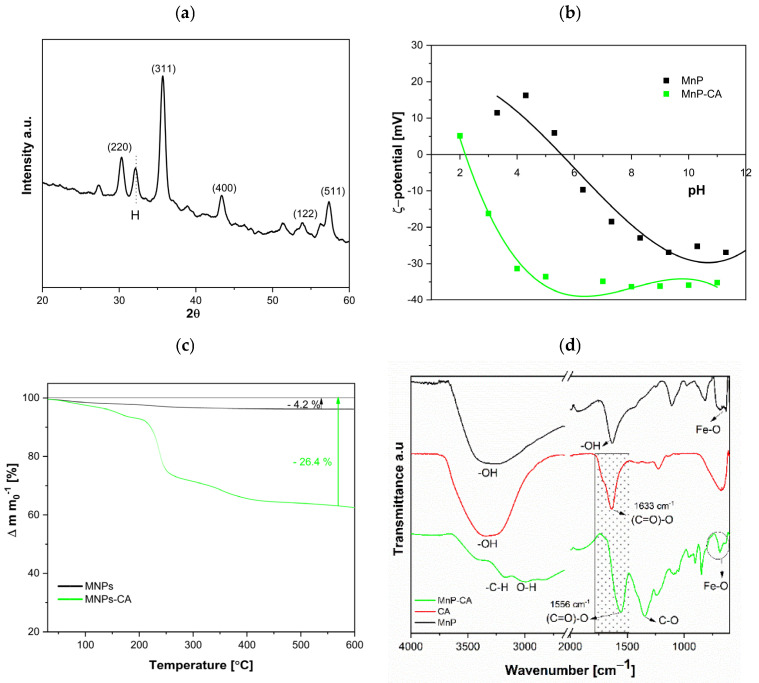
XRD spectrum of MnPs nanoparticles (**a**). The reflections were indexed based on the spinel structure. (H corresponds to the hematite); The ζ-potential as a function of pH for MnPs aqueous suspension and MnP-CA suspension (**b**). TGA curves for the MnP and MnP-CA nanoparticles (**c**). The ATR-FTIR spectra of MnP, MnP-CA nanoparticles and CA (**d**).

The properties of nanoparticles, such as their structure and surface characteristics, need to be tailored to meet the specific requirements of thermoset plastic (nano) composite applications. Nanoparticles typically have a tendency to agglomerate. However, when molecules adsorb onto the surfaces of nanoparticles, a surface charge is generated, creating repulsive forces that prevent agglomeration and make specific functional groups available for further interaction with the polymer. To illustrate the influence of nanoparticle integration on bio-EP composite properties, their crystallinity, zeta potential, and thermal and structural analyses are shown in Figure 2a–d.

Figure 2a displays the XRD patterns of iron-oxide nanoparticles synthesized via the coprecipitation of Fe^3+^ and Fe^2+^ ions from an aqueous solution using aqueous ammonia. The broad peaks correspond to the spinel ferrite structure, specifically maghemite (γ-Fe_2_O_3_). It is generally accepted that during the coprecipitation of Fe^3+^/Fe^2+^ ions, maghemite forms due to the oxidation of the resulting nanoparticles [40,44]. The XRD patterns of iron oxide nanoparticles at 2θ of 30.3, 35.7, 43.3, 53.8, and 57.3 are determined to be at the (220), (311), (400), (122), and (511) crystallographic planes of the spinel ferrite structure. Based on the broadening of the XRD reflections, the average crystallite size calculated from the Scherrer equation, was estimated to be 10.8 nm, respectively. While XRD cannot easily distinguish between magnetite (Fe_3_O_4_) and maghemite (γ-Fe_2_O_3_) due to their similar spinel structures, the oxidative synthesis conditions (presence of air, elevated pH, and aging) strongly favor the formation of maghemite over magnetite. Using CA as a stabilizer alters both the colloidal and surface properties of the nanoparticles. The ζ-potential of colloidal suspensions of magnetic nanoparticles (MNPs) and CA-coated MNPs (MNPs-CA) reflect changes in their surface behavior. Figure 2b illustrates how the ζ-potential varies with pH for both MNPs and MNPs-CA suspensions. The MNPs exhibit an isoelectric point (IEP) near pH 6, where the net surface charge is zero, leading to zero-reduced electrostatic repulsion and a tendency for particle aggregation. Upon coating with citric acid, the surface characteristics change significantly. CA molecules absorb onto the nanoparticle surfaces at acidic pH values, shifting the IEP from a neutral pH for MNPs (IEP = 6) to a strongly acidic value, around pH 2 for MNPs-CA. This shift is indicative of the strong interaction between CA and the nanoparticle surface, which alters the surface charge distribution. The CA-coated nanoparticles show a highly negative ζ-potential in the neutral pH region (approximately –40.5 mV at pH 6) compared to the less negative ζ-potential of uncoated MNPs in the same pH range. The pronounced negative ζ-potential of MNPs-CA demonstrates enhanced electrostatic repulsion, which contributes to colloidal stability and prevents particle aggregation. This stabilization is crucial for applications requiring well-dispersed nanoparticle suspensions in aqueous environments [1]. The observed differences in ζ-potential between MNPs and MNPs-CA confirm the formation of an adsorbed CA layer on the nanoparticle surfaces. This adsorbed layer not only alters the surface charge but also enhances the stability of the suspension by introducing strong repulsive forces. To further quantify the amount of CA on the MNPs, thermogravimetric analysis (TGA) measurements were conducted [40,45,46].

The comparative TGA results for MnP and MnP-CA, shown in Figure 2c, provide insight into their distinct thermal behaviors. The MnP nanoparticles exhibit a single, significant weight loss event above 100 °C, attributed to the desorption of chemically bound water. This observation aligns with findings reported for similar nanoparticles in the literature [39,47]. In contrast, the TGA results for MnP-CA nanoparticles show a more complex thermal profile. Weight loss occurs over a broader temperature range, from approximately 200 °C to 550 °C, with the most rapid decrease around 250 °C. The total weight loss for MnP-CA above 100 °C is about 25%, after which the weight loss stabilizes gradually beyond 550 °C. These results strongly suggest that citric acid (CA) is adsorbed onto the MnP nanoparticle surface, forming a protective layer that enhances thermal stability.

The presence of CA on MnP-CA nanoparticles is further confirmed by ATR-FTIR analysis, as shown in Figure 2d. This technique provides detailed information on the chemical structure of the nanoparticles and their surface modifications. The successful adsorption of CA onto MnP nanoparticles is evidenced by key characteristic peaks observed for both MnP and MnP-CA. Specifically, Fe-O bond vibrations, corresponding to both tetrahedral and octahedral sites, are detected in the range of (620 to 550) cm^−1^, indicating the integrity of the iron-oxide structure within the nanoparticles. Additionally, the presence of hydroxyl (OH) groups from adsorbed water is confirmed by distinct vibrational peaks at 3411 cm^−1^ and 1623 cm^−1^. These peaks are characteristic of water molecules interacting with the nanoparticle surface, emphasizing the role of surface-bound water in the colloidal stability of the nanoparticles [48]. In the ATR-FTIR spectrum of MnP-CA, a significant band at 545 cm^−1^, corresponding to Fe-O vibrations, indicates the successful adsorption of CA onto the MnP surface. This adsorption is crucial as it not only modifies the surface properties of the nanoparticles but also enhances their colloidal stability by preventing aggregation. Moreover, the splitting of the carboxyl band into two additional peaks at approximately 1570 cm^−1^ and 1370 cm^−1^ suggests the formation of a Fe-CA complex on the nanoparticle surface. This complexation likely results from the interaction between the carboxyl groups of CA and the iron atoms in the MnP structure. The formation of this complex is important because it confirms the strong binding of CA to the nanoparticles, which has implications for their stability, reactivity, and potential applications in various fields such as catalysis, drug delivery, and environmental remediation.

Overall, the combination of TGA and ATR-FTIR analyses demonstrates that CA significantly modifies the thermal and surface properties of MnP nanoparticles. This modification enhances their thermal stability and alters their surface chemistry, paving the way for potential applications that require stable, well-dispersed nanoparticles with specific functional properties.

**Figure 3:**
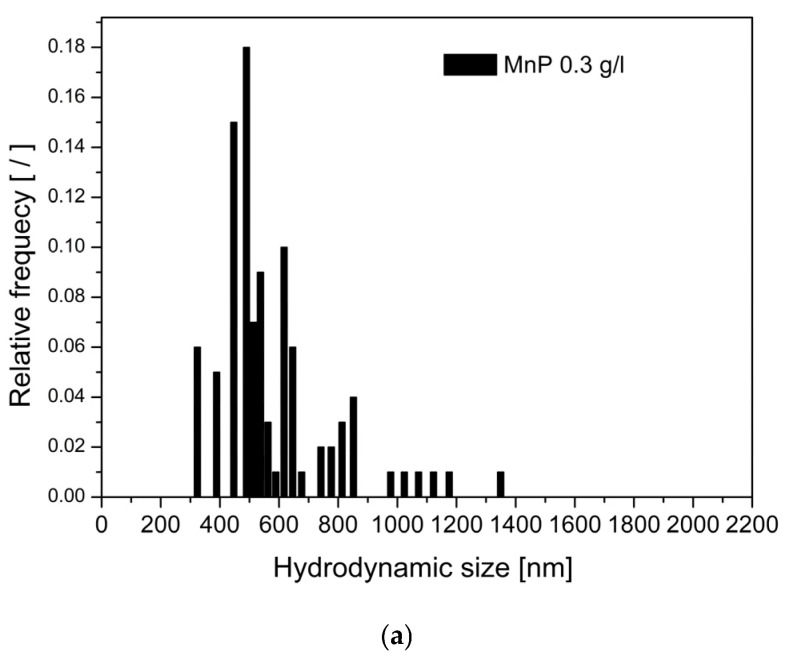

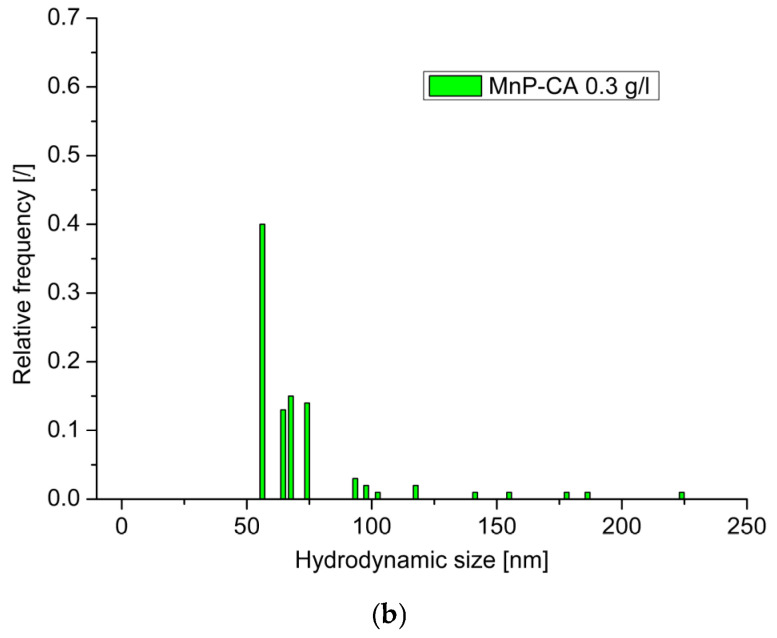
Hydrodynamic size distribution of MnP (**a**) and MnP-CA nanoparticles measured by dynamic light scattering (DLS) (**b**).

To evaluate the hydrodynamic size and size distribution of the MnP nanoparticles, dynamic light scattering (DLS) measurements were conducted for both uncoated MnP and citrate-coated MnP (MnP-CA) suspensions at a concentration of 0.3 g/L (Figure 3). The DLS results for the uncoated MnP nanoparticles (Figure 3a) show a broad size distribution, with hydrodynamic diameters ranging predominantly between 350 nm and 800 nm and some particles extending up to ~2000 nm. The distribution indicates significant aggregation in the suspension, with a peak relative frequency observed around 500 nm. In contrast, the MnP-CA nanoparticles (Figure 3b) exhibited a much narrower and more uniform size distribution. The majority of the particles had hydrodynamic diameters between 40 nm and 100 nm, with the most frequent size centered around 50–60 nm. This suggests that citrate coating significantly reduced aggregation, resulting in a more stable and monodisperse suspension. These findings confirm that surface modification with citrate improves colloidal stability and reduces nanoparticle aggregation, as evidenced by the decrease in average hydrodynamic size and the narrower distribution profile.

### 3.2. EP-MNP, EP-MNP-CA Characterization

To further quantify structural differences by identifying functional groups introduced or altered by the addition of nanoparticles in EP-MnP and EP-MnP-CA between neat bio-EP and EP-MnP resin composites, Attenuated Total Reflectance Fourier Transform Infrared Spectroscopy (ATR-FTIR) was employed. This technique effectively monitors changes resulting from nanoparticle incorporation into bio-EP resins, which subsequently enhance their final properties as well. ATR-FTIR enables detailed observation of chemical changes and functional group modifications arising from nanoparticle interactions within the bio-EP matrix. This understanding is crucial for developing advanced materials with tailored properties for specific applications.

**Figure 4:**
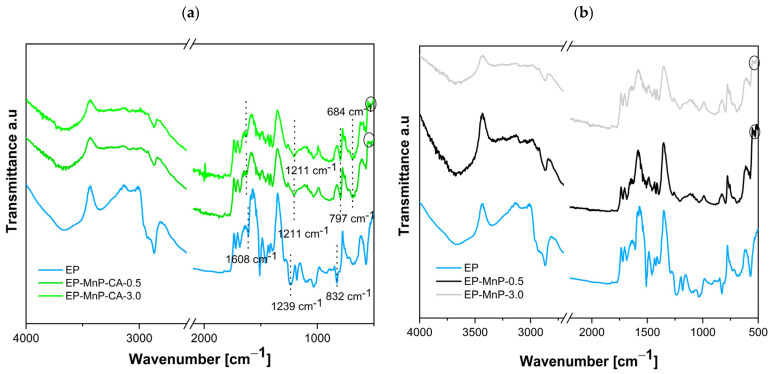
ATR-FTIR spectra of (**a**) EP-MnP-CA and (**b**) EP-MnP resin composites filled with 0.5 wt% and 3.0 wt% of nanoparticles.

The ATR-FTIR analysis shown in Figure 4a,b presents the spectra of neat bio-EP resin as well as all EP-MnP and EP-MnP-CA composites. The results of the ATR-FTIR analysis for neat bio-EP are given in Figure 4a. A detailed assignment of the epoxy ATR-FTIR spectra is listed in Table 3 [11,17,18,19] as well. The range at 3660 cm^−1^ and 3401 cm^−1^ corresponds to the overlapping –NH_2_ and –OH stretching vibrations [16]. Additionally, the ATR-FTIR spectra of the bio-EP display a broad band at 3401 cm^−1^ and 1608 cm^−1^, attributed to N–H stretching and N–H bending, respectively. The bands at 2966 cm^−1^ and 2928 cm^−1^ represent asymmetrical C–H stretching for CH_3_ and CH_2_ groups, while bands at 1511 cm^−1^ and 1390 cm^−1^ indicate CH_3_ deformation, and the band at 1460 cm^−1^ corresponds to CH_2_ deformation. The aromatic nature of the epoxy is clearly visible in the structure, and ring substitution can be assessed within the (1300–1000) cm^−1^ region when the epoxy resin chemically reacts with the hardener. Aldehyde C–H stretching appears at 2868 cm^−1^, while C–O stretching is observed at 1330 cm^−1^ [20]. The curing reaction between the epoxy and the amine group of the hardener is further supported by the disappearance of the peak at 918 cm^−1^, which is characteristic of the epoxide ring in uncured epoxy resin, as reported here [21].

Figure 4a,b presents the ATR-FTIR spectra for bio-EP containing 0.5 wt% and 3.0 wt% of MnP and MnP-CA, respectively. The incorporation of MnP-CA into the bio-EP resin is confirmed by a distinct vibration present in both the neat bio-EP and the MnP-CA composite. In the EP-MnP-CA-0.5 and EP-MnP-CA-3.0 samples, variations in the intensity and/or shifts in the characteristic vibration peaks are observed compared to the neat bio-EP. Common peaks in the EP-MnP-CA-0.5 and EP-MnP-CA-3.0 composites include 3660, 3340 cm^−1^ (O–H, N–H stretching), 2966, 2882, 2868 cm^−1^ (CH_2_ asymmetric and symmetric stretching), 1722, 1684 cm^−1^ (C=O stretching), 1511 to 1390 cm^−1^ (C–H deformation, CH_2_), 1293 cm^−1^ (C–O stretching), 1179, 1080, 1033 cm^−1^ (C–H bending), and 832, 532 cm^−1^ (C–H and O–H deformation). The presence of characteristic vibrations for MnP-CA at 684 cm^−1^ and peaks below 600 cm^−1^ confirms the successful incorporation of MnP-CA in the EP-MnP-CA composites (sign with circle in Figure 4a). In the ATR-FTIR spectra of MnP-CA, the appearance of new, intense absorption peaks at 1552, 1348, and 1244.6 cm^−1^ indicates the formation of carboxylate groups from citric acid, which complex with Fe atoms on the maghemite surface, as reported in the previous chapter (Figure 2d). Here, in the EP-MnP-CA composites, the N-H group of the bio-EP reduces the intensity of the characteristic vibration at 1608 cm^−1^ in EP-MnP-CA-0.5 and EP-MnP-CA-3.0 samples. Additionally, the carbonyl absorption frequency is shifted from 1238 to 1211 cm^−1^ (Figure 4a), suggesting the formation of intermolecular hydrogen bonds between MnP-CA particles and the bio-EP matrix,and suggesting strong intermolecular hydrogen bonding. Conversely, the spectra of bio-EP containing unmodified MnP particles, which resemble those of neat bio-EP and neat MnP (see Figure 4b), show no notable changes in peak formation, with peaks characteristic of MnP nanoparticles, such as Fe–O near 530 cm^−1^, remaining unchanged (Figure 4b, sign with circle).

The ATR-FTIR analysis was conducted to assess potential bonding between MnP-CA and bio-neat EP, arising from the interaction of nanoparticles within the bio-EP resin. Additionally, to evaluate the impact of nanoparticle interactions on the thermal properties and stability of all EP-MnP and EP-MnP-CA composites, thermogravimetric analysis (TGA) was performed at temperatures reaching up to 800 °C.

**Figure 5:**
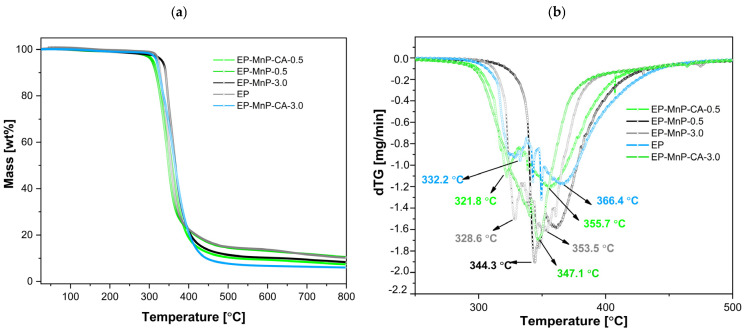
TGA thermograms (**a**) and dTG curves (**b**) of neat bio-EP resin, and all EP-MnP-0.5, EP-MnP-3.0, EP-MnP-CA-0.5, EP-MnP-CA-3.0 resin composites.

The obtained results are illustrated in Figure 5a, b and represent the weight loss curves in relation to the temperature, with the peak/decomposition temperature (Td). These processes contributed to further degradation during the second stage of weight loss, which occurred between 200 °C and 400 °C. The successful removal of trace water and solvents was attained during fabrication, as no weight loss was found below 120 C. At around 300 °C to 400 °C, degradation began gradually through the homolytic cleavage of chemical bonds in the ester linkages within the network, triggered by the dehydration of the oxypropylene group, –CH_2_-CH(OH), leading to the formation of double bonds. The decomposition temperature (Figure 5b) for neat bio-EP resin was approximately (332.67 ± 0.25) °C. In this initial phase, isomerization reactions, chain transfers, intermolecular cyclization, and other radical reactions were triggered, marking the first stage of degradation. For bio-EP resins, thermal degradation typically involves the breakdown of its crosslinked network. Bio-based components, such as bio-derived hardeners or curing agents, often decompose at higher temperatures due to their complex chemical structures. This process includes the degradation of bio-based polyols or other functional groups derived from renewable sources, contributing to the material’s overall weight loss. As the degradation proceeds into the second stage, at 366.1 °C, further weight loss occurs due to the complete breakdown of the polymer backbone. This involves the scission of additional chemical bonds in both the epoxy network and bio-based additives. The release of volatile compounds and char formation is typical in this stage and depending on the specific formulation of the bio-epoxy resin, the thermal degradation may also involve reactions like oxidation or decomposition of remaining bio-functional groups. Bio-epoxy resins thermally degrade through a multi-stage process. In our samples, we performed a two-stage process, starting with bond cleavage and leading to the breakdown of both synthetic and bio-based components, with subsequent weight loss and the formation of volatile products.

The addition of nanoparticles, whether pristine or modified, results in an enhancement of the bio-EP resin’s thermal stability obtained from DTG curves (Figure 5a,b), confirming changes in mass over a wide range of temperatures. In general, the degradation starts at around 300 °C, indicating a shift in thermal stability depending on the surface modification and its concentration. As shown in Figure 5a,b, the Td for the EP-MnP-0.5 composites dips slightly below the benchmark, reaching a maximum of one peak of 344.2 °C (around 16 °C less than neat bio-EP), proving the increased average degradation temperature of EP-MnP-0.5 resin composites compared with neat bio-EP. With an increasing MnP loading content (3.0 wt%), the Td of the first peak was unretained at 328.62 °C, while the second peak appeared at 353.1 °C. In the subsequent temperature range, the composites underwent secondary decomposition between 400 and 500 °C, attributed to the further breakdown of the nanoparticle/epoxy/hardener network [20,49].

When reinforced with MnP-CA, the decomposition appeared in two stages, similar to neat bio-EP, but due to the MnP-CA concentration, it provided differences in the degradation T. The weight loss associated with the degradation of EP-MnP-CA-0.5 composites began at lower temperatures (a minor one at around 321.8 °C) and continued through the next stages up to higher temperatures, with a second degradation peak temperature at 355.1 °C, indicating multiple degradation phases influenced by the presence of nanoparticles. Contrarily, the addition of 3.0 wt% of modified nanoparticles led to a higher degradation temperature at 347.6 °C, leading almost to a one-step degradation phase. Notably, the introduction of 3.0 wt% of functionalized iron-oxide based nanoparticles provided a more available surface functional group, thus leading to an increase in interaction between the bio-EP and nanoparticles, providing activation energy for thermal degradation, which suggests improved thermal stability.

The dispersion of MnPs in bio-EP can significantly improve the thermal stability of epoxy composites. Properly dispersed nanoparticles enhance heat transfer throughout the matrix, resulting in increased thermal conductivity and greater resistance to thermal degradation [50,51]. This effect is especially notable with smaller nanoparticles, as their larger surface area allows for greater interaction with the bio-EP matrix. According to the literature, incorporating nanoparticles can increase the thermal stability of these composites, owing to the existence of a rigid part of nanoparticles, which acts as an interdiction to minimize the permeability of volatile degradation products from the epoxy nanocomposites [52]. Enhanced thermal stability of EP-MnP and EP-Mnp-CA composites follow the effects studied in the literature due to synergistic effects, physical barriers, improved kinetics, and concentration effects [20,49,53,54].

In summary, the temperature of degradation for MnP- and MnP-CA-filled epoxy resins varies significantly based on the type and number of nanoparticles used. Generally, significant mass loss occurs between 300 °C and 480 °C, with complete degradation extending up to 700 °C. The incorporation of 0.5 wt% MnP and 3.0 wt% MnP-CA nanoparticles tends to improve thermal stability, shifting initial decomposition temperatures higher compared to unfilled bio-EP.

The heat evolution observed in epoxy resin using DSC is generally assumed to be proportional to the extent of reactive group consumption. The curing reaction of epoxy resin is a complex and critical process that transforms the liquid resin into a durable, solid thermoset material through a series of chemical reactions. Typically, the curing of epoxy resins involves a reaction between epoxy groups and curing agents—such as amines, anhydrides, or acids—which play a vital role in determining the crosslinking mechanism of the curing process [55,56]. These mechanisms may include ring-opening reactions, nucleophilic attacks, and crosslinking, all of which contribute to the development of a rigid, highly crosslinked network. Moreover, the curing reaction is temperature-dependent; higher temperatures generally accelerate the curing process.

Not just the selection of curing agents and curing conditions (such as temperature and time) but also the addition of nanoparticles and the overall composition of the resin system can significantly influence the curing kinetics and, consequently, the final properties of the cured material. The final factor—iron oxide nanoparticles—has a substantial impact on the curing reaction of epoxy resin, enhancing various properties, including the mechanical performance of the resulting composite material. The curing process, which involves the crosslinking of epoxy with a hardener, can be influenced by both the incorporation of bare and functionalized nanoparticles, as well as the concentration of nanoparticles used.Factors such as the degree of crosslinking and the resulting network structure affect the mechanical strength, thermal stability, chemical resistance, and durability of the epoxy. By carefully controlling the curing process, manufacturers can tailor the material properties to meet specific application requirements, making epoxy resins widely used in coatings, adhesives, electronics, and composites.

**Figure 6:**
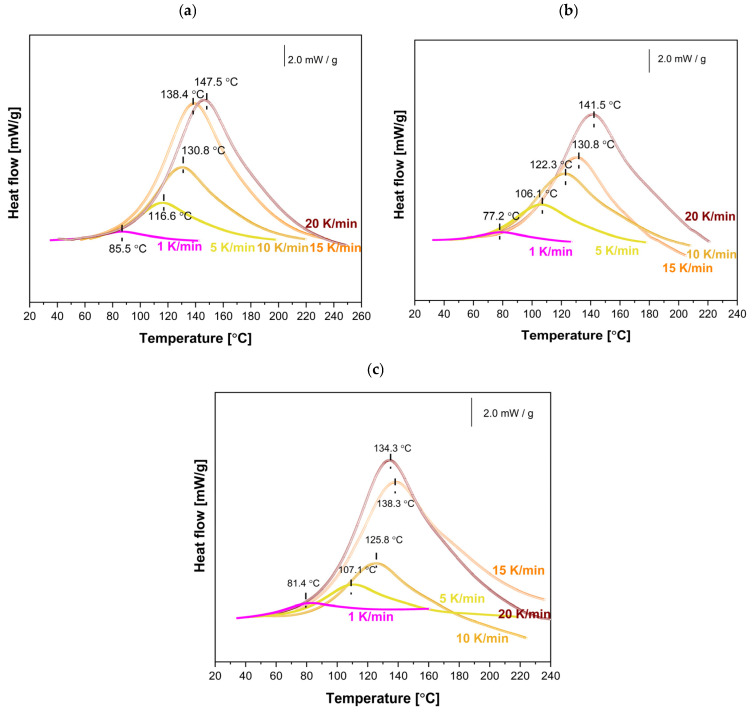
Non-isothermal DSC cure behavior of the (**a**) bio-EP resin, (**b**) EP-MnP-0.5 and (**c**) EP-MnP-3.0 are presented, with experimental data shown as symbols and the lines representing the best model fits.

**Figure 7:**
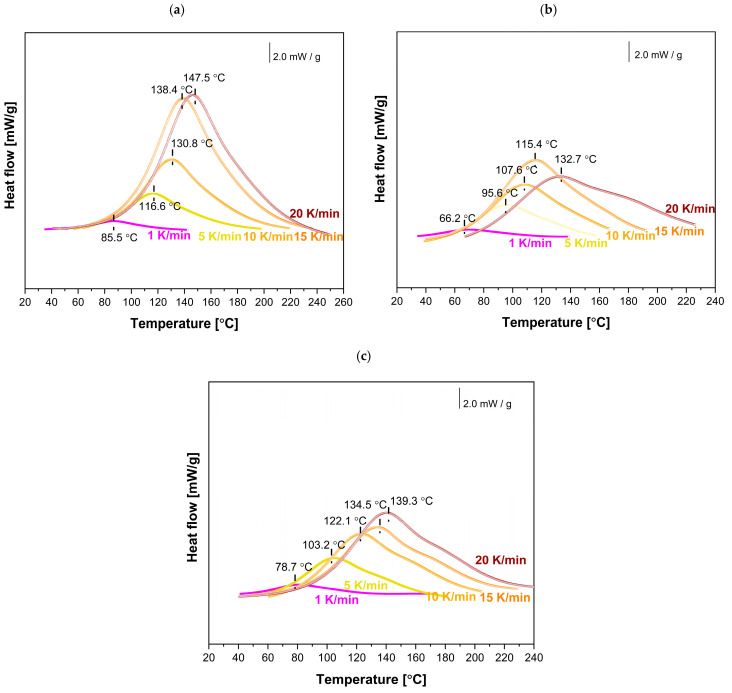
Non-isothermal DSC cure behavior of the bio-EP resin (**a**), EP-MnP-CA-0.5 (**b**), and EP-MnP-CA-3.0 (**c**) is presented, with the experimental data shown as symbols and the lines representing the best model fits.

To investigate the effect of MnP and MnP-CA nanoparticle addition on the curing reactions of the EP-MnP and EP-MnP-CA systems, the initial step involved selecting the optimal curing temperatures based on the analysis of dynamic cure DSC curves obtained from non-isothermal DSC measurements (Figure 6 and Figure 7).Subsequently, a series of measurements of the non-isothermal curing reactions at different heating rates, ranging from 1 to 20 K/min, was conducted (Figure 6 and Figure 7) for EP, EP-filled MnP, and EP-filled MnP-CA, which plots the heat flow (dH/dt) against temperature (T). The key information that can be directly extracted from the DSC curve includes the onset temperature (Ti), peak temperature (Tp), and the values of dH/dt.

For neat bio-EP, as expected (Figure 6 and Figure 7a), the DSC is determined by a broad exothermic peak, and with the increasing heating rate, the magnitude of the exotherm increases as well, and the temperature of the peak shifts to a higher temperature value due to the increasing heating rate.

Due to the MnP addition (0.5 wt% and 3.0 wt%), the heat flow is enhanced with the increasing heating rate, as shown in Figure 6b,c. It is evident from Figure 6b,c that even for the EP-MnP-0.5 epoxy resin composite or EP-MnP-3.0, the peak temperature of curing shifts. As the heating rate increases, the curing start temperature (Ti), peak temperature (Tp), and end temperature (Tf) shift to lower values compared to neat bio-EP. The results suggest that MnP acts as a trigger for the fastest curing process of EP-MnP composites. Thus, the % of the loading of MnP impacts the reaction kinetics of all EP-MnP composites. Higher heating rates tend to shift the curing start temperature (Ti), peak temperature (Tp), and end temperature (Tf) to lower values, indicating that the EP-MnP resin reacts more quickly at elevated temperatures than neat bio-EP, providing lower activation energy to be utilized in a shorter time frame.

When nanoparticles are functionalized with citric acid (CA), the curing process of all EP-MnP-CA epoxy resin composites is further enhanced compared to EP-MnP composites filled with bare iron oxide nanoparticles.. Figure 7 represents the comparison of curing temperatures of neat bio-EP, MnP-CA-0.5, and EP-MnP-3.0 resin composites by analyzing the dynamic cure DSC curves, a non-isothermal curing reaction at different heating rates ranging from 1 to 20 K/min focusing on the onset temperature (Ti), peak temperature (Tp), terminal temperature (Tf), and the values of dH/dt. It is evident that due to the incorporation of 0.5 wt% or 3.0 wt%, the temperature of curing is enhanced and shifted to the lowest value of temperature. More evident with the increasing content of nanoparticles is that the temperature of curing decreases. The critical factors influencing the curing process and the resulting material properties include the addition of nanoparticles and their interaction mechanisms. It is well known that, at low concentrations, these nanoparticles enhance the curing reaction due to their high surface area, which promotes better interaction with the epoxy matrix. They can act as catalysts in the curing process, promoting the ring-opening of epoxy groups and leading to a denser crosslinked network [30,38,57]. This is particularly effective when using amine-functionalized Fe_3_O_4_ nanoparticles, which actively participate in the curing reaction by attacking epoxy groups.

**Figure 8:**
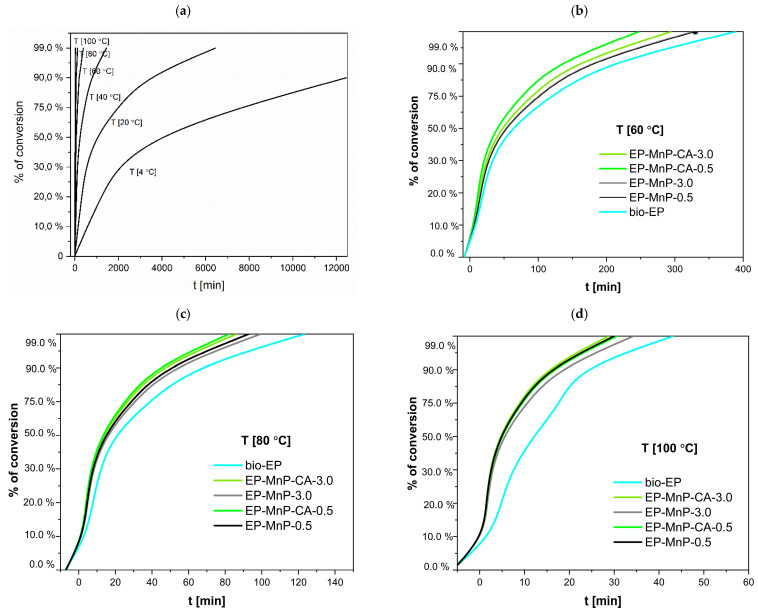
Fractional extent of reaction vs. time for the prepared bio-EP resin (**a**) and all EP-MnP, EP-MnP-CA resin filled with either 0.5 wt% or 3.0 wt% nP at different temperatures T = 60 °C (**b**), T = 80 °C (**c**) and T = 100 °C (**d**).

The dispersion of bare nanoparticles differs from that of surface-modified nanoparticles in the bio-EP resin, resulting in variations in the degree of curing that depend on the temperature heating rate, as shown in Figure 8 and Table 3. The curing degree of neat bio-EP increased rapidly during the initial reaction stage with the increasing temperature heating rate, then increased slowly, and finally tended to a certain value. Nevertheless, the time for curing the formulations loaded with MnP and MnP-CA greatly varies depending on the amount of MnP (Table 3). Nevertheless, this effect is more visible at low temperatures for neat bio-EP. The time needed for 99% curing conversion at room temperature, is 80.9 h. With an increasing temperature to 60 °C, the curing conversion decreases to shorten the time to 320.41 min, and with a further increasing temperature, at 100 °C, in only 50.98 min, 99% bio-EP-neat is cured. Compared with EP-MnP filled with 0.5 wt.% and 3.0 wt.% MnP, the curing conversion of all composites is fastened, providing less time for 99% of cured EP-MnP composite samples. The 0.5 wt% MnP-loaded nanoparticles exhibit the fastest curing conversion of EP-MnP composites compared to neat bio-EP at temperatures of 60 °C, 80 °C, and 99 °C, with curing times of 328.92 min and 29 min for EP-MnP-0.5, and 327.99 min and 34 min for EP-MnP-3.0, respectively. At higher heating rates, the conversion of curing curves becomes faster than that of neat bio-EP. This indicates that, due to the MnP addition, more heat is released during the curing process, which can enhance crosslinking.

The same trend of curing conversion behavior is evident for the MnP-CA 0.5 and MnP-CA-3.0 composites’ resin (Figure 8 and Table 4). With the incorporation of 0.5 wt% MnP-CA, the curing process is faster than in neat bio-EP and all EP-MnP composites. At 60 °C, the curing conversion is achieved in 247 minutes, approximately 100 minutes faster than neat bio-EP. At 100 °C, EP-MnP-0.5 reaches curing conversion in only 30 minutes. The addition of 3.0 wt% MnP-CA further accelerates the curing process, with conversion occurring at 294.86 °C and in 28 minutes. EP-MnP-3.0 also cures efficiently at 60 °C, 80 °C, and 99 °C.

The results shown in Figure 8 and Table 4 highlight several critical factors that influence the curing of bio-EP resin filled with bare and surface-modified MnP nanoparticles. The recent research findings focus on the following aspects. The incorporation of iron-oxide nanoparticles, bare or surface-modified, into bio-epoxy resin significantly influences its curing reaction and kinetics. The curing process, which involves the crosslinking of epoxy with a hardener, can be tuned: first, by the concentration of nanoparticles used; second, by the functionalization of surfaces of nanoparticles; third, by the dispersion in EP, resulting in an enhanced interaction between n EP and MnP, providing faster curing than neat bio-EP. At lower loadings (e.g., 1–5 wt%), the nanoparticles remain well-dispersed, enhancing the curing process without significant aggregation. This results in improved mechanical and thermal properties of the cured epoxy [38,57]. Bare Fe_2_O_3_ nanoparticles tend to agglomerate, especially at higher concentrations. This clustering leads to voids within the epoxy matrix, which can hinder effective crosslinking during the curing process. The tendency for bare nanoparticles to form clusters is attributed to van der Waals forces and magnetic interactions, which can deteriorate the mechanical properties and barrier performance of the cured epoxy. Surface-modified nanoparticles enhance curing kinetics by participating more effectively in crosslinking reactions due to their reactive functional groups acting as a curing promoter as well. This leads to a more effective curing process, especially at lower concentrations. Similar results found in the literature reveal that the surface modification (e.g., with SiO_2_ or amine groups) enhances the chemical stability and compatibility of Fe_3_O_4_ nanoparticles with the epoxy resin. This results in a more uniform dispersion throughout the matrix and enhanced interaction. Functionalized nanoparticles can participate in chemical interactions with the epoxy, promoting better integration into the matrix.This leads to a denser crosslinked network and fewer voids, improving mechanical properties and corrosion resistance. As reported in Ref. [57], the addition of bare iron oxide nanoparticles (particularly Fe_3_O_4_, magnetite) at low contents of nanoparticles has been shown to decrease the curing enthalpy of epoxy systems. This suggests that the presence of these nanoparticles can facilitate the curing reaction, potentially leading to a more efficient crosslinking process, demonstrating a similar trend in our study when MnP-CA is incorporated in bio-EP composites’ resin. Additionally, the published work investigated the effect of bare, SiO_2_/chitosan-, and SiO_2_/chitosan/imide/phenylalanine-modified Fe_3_O_4_ nanoparticles in EP resin, demonstrating a curing-promoting effect. This effect was more pronounced in samples where Fe_3_O_4_; was functionalized with chitosan [38]. Previous studies have shown that nanoparticle chemistry significantly affects curing behavior. For example, Co^2+^-doped Fe_3_O_4_ was reported to increase the heat released during epoxy curing, while AuNPs were found to reduce the exothermic response—likely due to steric hindrance effects. These findings support the notion that nanoparticle composition and surface reactivity play a key role in tailoring crosslinking kinetics, although such doped systems were not part of the present study. Even more, it is clear that nanoparticles play a dual role as curing promoters, resulting in increased viscosity of the system and leading to the gelation process due to uniform dispersion of nanoparticles in bio-EP resin.

Dynamic mechanical properties (DMA) of bio-based EP resin composites filled with the nanoparticle filler, including storage modulus, loss modulus, and glass transition, depend on the addition of either neat or functionalized fillers and provide crucial knowledge for applications requiring enhanced mechanical performance and thermal stability. Here, the morphology and size of the fillers play a crucial role in determining the mechanical properties, where the smaller particles more uniformly distribute in the bio-EP resin matrix, leading to more sufficient reinforcement and minor agglomeration [58,59,60]. It is well established that the DMA properties of epoxy-based nanocomposites vary depending on the type of filler, its surface modification, and temperature. These factors influence the viscoelastic behavior of the composites. Moreover, the incorporation of fillers into bio-based epoxy generally leads to an increase in storage modulus, indicating enhanced stiffness and load-bearing capacity.

**Figure 9:**
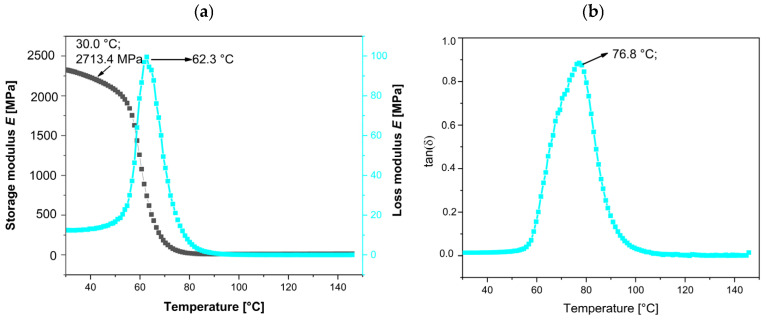
Storage modulus (**a**) and tan delta (**b**) of bio-EP.

The neat bio-EP resin, as a thermoset material, exhibits a high storage modulus (E′), loss modulus (E″), and damping factor (tanδ), as shown in Figure 9a,b. The initial values of E′, E″ at the peak, and maximum tanδ are summarized in Table 5. At 25 °C, the initial E′ value is 2710.4 MPa, while the tan delta (tanδ), representing both the elastic and viscous components of this viscoelastic polymer, peaks at 76.8 °C.

**Figure 10:**
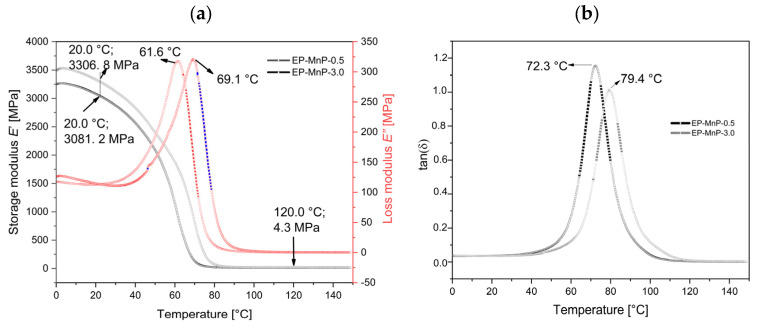
Storage modulus and Loss modulus (**a**) and tan delta (**b**) of EP-MnP composites filled with 0.5 and 3.0 wt.% of filler content.

**Figure 11:**
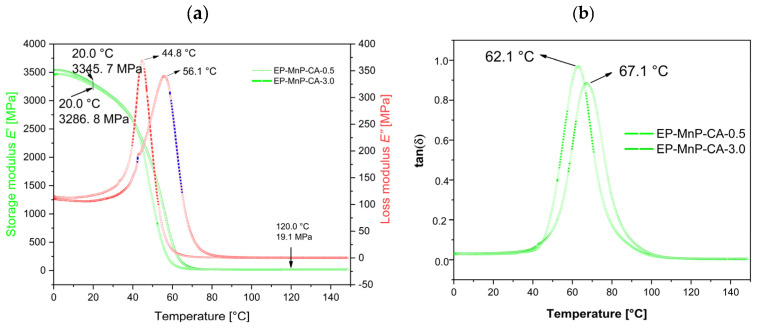
(**a**) Storage modulus, Loss modulus and (**b**) tan delta of EP-MnP-CA filled with 0.5 and 3.0 wt.% filler content.

The evolution of storage modulus (E′) in MPa, loss modulus (E″) in MPa, and tangent delta (tanδ), or loss factor/damping factor (non-dimensional) for the EP, EP-filled MnP, and MnP-CA samples is summarized in Figure 9, Figure 10 and Figure 11 and Table 5 to further illustrate the dynamic mechanical properties. Figure 10 shows the effect of different MnP loadings (0.5 and 3.0 wt%) on the storage modulus, loss modulus, and tan delta of all EP-MnP composites. Adding either 0.5 or 3.0 wt% of MnP enhances the storage modulus of EP composites at 20 °C: a 14.1% increase with 0.5 wt% MnP and a 24.7% increase with 3.0 wt% MnP, as shown in Figure 10a,b and summarized in Table 5. The addition of rigid MnP particles makes the EP-MnP composites tougher and stiffer than the neat epoxy composites. Thus, Figure 10 clearly shows that the tensile strength of the epoxy composites improves with the addition of an MnP filler. This enhancement in storage modulus is attributed to the reinforcing effect of MnP particles, which also results in changed mobility of the EP-MnP composites, as indicated by the glass transition temperature (Tg). Additionally, the ratio of the elastic to viscous portions in EP-MnP composites, represented by tanδ, is shown in Figure 10b. With increasing MnP content, the Tg value initially rises to 79.4 °C for the 0.5 wt.% MnP addition but slightly decreases to 72.1 °C at 3.0 wt% compared to the neat bio-EP material. This increase in Tg is likely due to enhanced movement within the EP-polymer network chains caused by the nanoparticle addition, which leads to the formation of free volume that restricts the chain’s mobility, as it was found in ZnO-EP composites and Fe_3_O_4_/GPTMS/Epoxy composites.

On the other hand, the storage modulus, loss modulus, and tan delta of all EP-MnP-CA composites, as shown in Figure 10a and Figure 11a,b, exhibit higher values compared to both neat bio-EP and EP-MnP composites. Specifically, as the content of modified nanoparticles increases from 0.5 wt% to 3.0 wt%, the storage modulus of the EP composites increases by 20.1% and 22.4%, respectively. This indicates the successful transfer of applied stresses from the polymer matrix to the reinforcement effect. As can be seen in Figure 11a, all the samples for EP-MNP-CA, independent of the proportion, present slight increments in the rigidity (storage modulus) in both glassy (20 °C) and rubbery states (120 °C).

The tan delta of EP-MnP-CA composites was also determined. It was observed that the Tg notably decreases when the EP is reinforced with modified nanoparticles. The results summarized in Table 5 and Figure 11b indicate an increase in Tg from 62.2 °C for neat bio-EP to 67.1 °C for EP-MnP-CA-0.5 composites and further for EP-MnP-CA-3.0 composites, demonstrating the enhanced thermal performance with increasing MnP-CA content. It was also observed that the height of the tanδ peak (which is related to the mobility of the network structure) changes when increasing the proportion of 0.5 wt% and decreases to 3.0 wt% (Table 5). Similar results have reported a similar trend, with none of the proposed theories being conclusive. In a study on EP-based magnetite nanoparticle composites, when functionalized with either PDA, GPTMS, or APTES on maghemite nanoparticles, the tan delta (Tg) shifted to a lower temperature. It has been suggested that the interaction between the polymer matrix and the reinforcements leads to the formation of multiple layers around the particles. A secondary polymer nanolayer that forms around the nanoparticles is believed to relax more quickly than the immobile layer directly bound to the NPs. This portion of the matrix can move more freely at higher temperatures, potentially reducing the composite’s Tg [61]. Another study on EP–organoclay composites found that chain entanglement and excess free volume within the matrix system lead to a decrease in Tg values. This reduction is associated with increased molecular mobility, which in turn lowers the reaction rate acceleration and effective crosslink density, ultimately reducing the curing temperature [18].

To summarize, the reinforcing effect of either neat or functionalized nanoparticles is attributed to improved storage modulus and is more pronounced in functionalized nanoparticles. First, the functionalizing of nanoparticles is crucial for optimizing the interaction with the epoxy matrix, leading to better dispersion and load transfer, which also restricts macromolecular mobility near the nanoparticles. It is clearly measured that as the nanoparticles were further modified, Tg decreased to lower values. This decrease in Tg is a clear indication of hindrance in more uniformly distributed nanoparticles that provide better adhesion of nanoparticles in the epoxy matrix, which may result in restricted chain mobility. However, when unmodified MnP is incorporated into EP composites, it does not significantly hinder dispersion, initiate agglomeration, or cause notable changes in the Tg value.With respect to the storage modulus in the glassy and rubbery state, the tendency is similar to the peak of tanδ. As the nanoparticles are modified, the storage modulus at 120 °C reaches 14.4 MPa compared to non-modified MnP nanoparticles, which decreases the storage modulus to 4.4 MPa. The results of the storage modulus at the rubbery state indicate better distribution in the polymer matrix and pronounced crosslinking density. The storage modulus in the rubbery state is also related to the crosslinking density, reaching the highest value at 120 °C at 19.1 MPa when the nanoparticles are modified with CA, and 4.3 MPa when EP-composites consist of non-modified nanoparticles [18]. This highlights the advantageous role of nanoparticle functionalization, which introduces polar groups—such as hydroxyl and carboxyl—on the nanoparticle surface, thereby enhancing their interaction with the epoxy matrix. The modified MnP-CA nanoparticles provide these functional groups and play a critical role in improving the properties of epoxy composites through multiple mechanisms, primarily involving enhanced interfacial interactions and physical reinforcement. It also allows for the formation of hydrogen bonds between the filler and the epoxy matrix and creates an interface between bio and EP. These interactions are crucial for enhancing filler–matrix adhesion, which is essential for transferring stress effectively during mechanical loading. Stronger interfacial bonding leads to improved tensile properties and overall durability of the composite. In addition, hydroxyl groups in fillers can catalyze the polymerization process of epoxy resins curing. This catalytic effect facilitates the diffusion of epoxy and curing agent molecules, leading to improved crosslinking within the matrix. Enhanced crosslinking results in a more robust network structure, contributing to higher mechanical strength and thermal stability of the composite. In the literature, it was also known that fillers with hydroxyl functional groups can significantly enhance the mechanical properties of epoxy composites. For instance, EP composites reinforced with silicate nanofillers containing hydroxyl groups exhibited increased tensile strength and storage modulus compared to those without such functionalization. This enhancement is attributed to better dispersion and interaction at the filler–matrix interface [50,62,63]. Although the functionalization of MnP nanoparticles with CA enhances dispersion and filler–matrix interactions, the observed decrease in Tg for EP-MnP-CA composites may be attributed to a plasticization effect induced by the hydrophilic functional groups (–OH, –COOH) of citric acid. These groups can increase local free volume or form loosely bound interfacial regions with greater chain mobility. Similar behavior has been reported in epoxy systems functionalized with organic modifiers, where improved dispersion is often accompanied by a reduction in Tg due to enhanced segmental motion in interfacial zones [62]. Additionally, variations in local curing behavior and crosslinking density near the functionalized nanoparticle surfaces may further contribute to this effect. 

The incorporation of iron-oxide nanoparticles, specifically magnetite (Fe_3_O_4_) and hematite (Fe_2_O_3_), into EP composites not only affects the curing process, providing differences in thermal properties, but significantly influences their surface properties as well. In the following study, the surface properties of bio-EP, EP-MnP, and EP-MnP-CA were evaluated with surface zeta potential measurements, resulting in differences in the surface charge.

Surface charge plays an essential role in describing how EP-based nanocomposites interact with other materials in aqueous environments, particularly solid surfaces. This charge characteristic becomes critical for understanding how bio-EP resin composites behave before and after introducing iron-oxide-based fillers. The addition of such fillers can significantly alter the material’s interfacial properties, compatibility, and potential uses in various applications, such as biomedical devices, aerospace, automotive, construction, and coatings. To explore these changes, zeta potential measurements were conducted, targeting the surface properties of neat EP composites while varying the type and concentration of bare MnP and MnP-CA. These measurements provide a quantitative view of the surface charge across a pH range, enabling a better understanding of how different fillers influence composite surface behavior.

**Figure 12:**
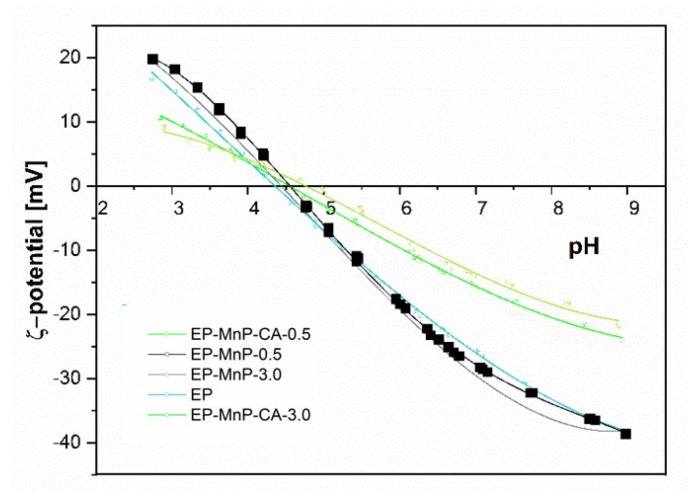
Zeta potential of bio-EP resin composite samples filled with 0.5 wt%, 3.0 wt% MnP and MnP-CA measured in the range of pH 2–10.

Figure 12 presents the surface zeta potential values for neat bio-EP, EP-MnP, and EP-MnP-CA composites across a pH spectrum from 2 to 12, demonstrating how MnP filler additions shift the surface charge. These zeta potential and isoelectric point (IEP) values reveal essential details about the functional groups on the composite surface. For instance, the isoelectric point serves as a valuable indicator of surface acidity or basicity, with a lower IEP suggesting an increase in acidic functional groups, while a higher IEP points toward additional basic groups. As shown in Figure 12, the IEP for neat EP occurs at a pH of approximately 4.3, a result that aligns with existing literature on EP resin composites. Interestingly, with the incorporation of MnP and MnP-CA fillers, the isoelectric point (IEP) remains relatively unchanged across different concentrations, showing only slight variations: EP-MnP-0.5 = 2.90, EP-MnP-3.0 = 3.05, EP-MnP-CA-0.5 = 3.21, and EP-MnP-CA-3.0 = 3.21. For instance, the IEP values for EP-MnP and EP-MnP-CA composites range from pH 4.5 to pH 4.7, indicating a slight shift toward the right (less acidity) but overall stability in the IEP. The stable IEP values suggest that the additions of MnP and MnP-CA do not significantly alter the fundamental surface charge properties of the EP matrix. This modest change suggests that the filler particles are not significantly altering the surface chemistry of the composites, likely because they are embedded within the epoxy matrix rather than being predominantly present at the surface. The hydrogen bonding or other interfacial interactions (e.g., with carbonyl groups of EP reported by ATR-FTIR results) between the fillers and the matrix might also contribute to this observation. These interactions stabilize the filler within the matrix without dramatically altering the external surface charge properties.

This negligible effect on the surface charge results in a relatively consistent IEP value, highlighting the compatibility and cohesive interaction of EP with fillers.

The overall zeta potential of EP-MnP and EP-MnP-CA composites, which shift from high negative values at an elevated pH towards more positive values, is shown in Figure 12. Above a pH of 4.7, all EP-MnP and EP-MnP-CA composites exhibit increasingly negative zeta potentials. The impact of MnP and MnP-CA incorporation on the surface and interfacial charge of these composites is reflected in the negative zeta potential values at a pH of 8 (Figure 12). This may primarily reflect charge redistribution within the matrix rather than a wholesale alteration of surface functionality. However, although no significant changes in IEP occurred, it is evident that as MnP-CA is incorporated into EP, the rise in the negative zeta potential plateau values occurred. With higher levels of MnP-CA, the zeta potential at a pH of 8 becomes more negative than that of neat EP. This effect is attributed to the addition or redistribution of polar groups on the composite surfaces, which enhances surface hydrophilicity. Overall, zeta potential measurements for all EP-MnP and EP-MnP-CA composites exhibit an anionic charge above their respective IEP values, indicating a high capacity for adhesion or adsorption of cationic substances and the repulsion of anionic substances. Nevertheless, the incorporation of CA-functionalized MnP (MnP-CA) results in a more negative ζ-potential at neutral-to-basic pH values compared to neat EP and EP-MnP composites. This behavior can be attributed to the presence of ionizable carboxyl and hydroxyl groups introduced by citric acid on the nanoparticle surface. These groups enhance the hydrophilicity and polarity of the filler surfaces and facilitate stronger interfacial interactions (e.g., hydrogen bonding) with the epoxy matrix. Although the isoelectric point remains relatively unchanged—likely due to the encapsulation of fillers within the matrix—the increase in a negative surface charge above the IEP confirms that functionalization leads to a redistribution of surface-active groups and improved matrix compatibility.

**Figure 13:**
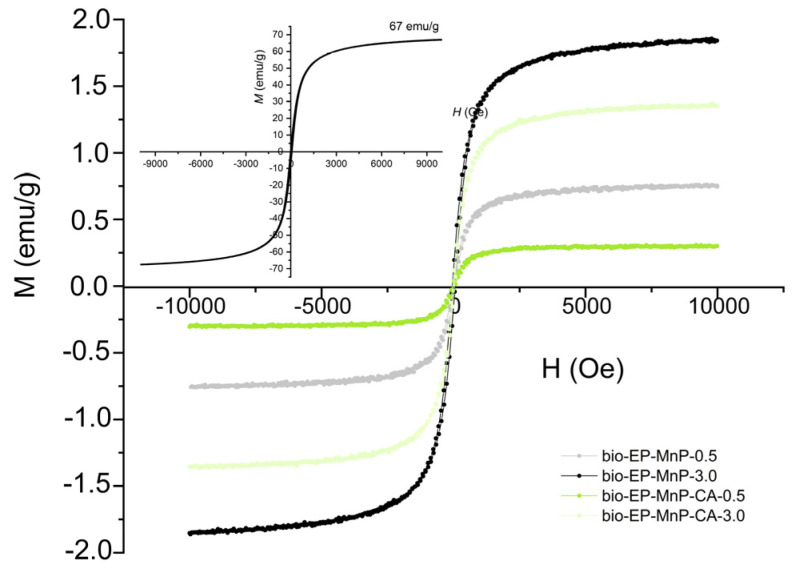
Response of superparamagnetic iron oxide nanoparticles in bio-EP composites in an external magnetic field.

The incorporation of MNPs is also significant for the practical applicability of magnetic bio-epoxy resin composite materials. The iron oxide nanoparticles, synthesized via coprecipitation with an appropriate ratio of precursor salts (iron sulfates), exhibit a structural composition that is a mixture of maghemite (γ-Fe_2_O_3_) and magnetite (Fe_3_O_4_). These nanoparticles are characterized by superparamagnetic properties, which depend on their particle size. While the magnetic moments of individual atoms remain aligned, the overall magnetic moment of the nanoparticle fluctuates rapidly under the influence of thermal energy. In the absence of an external magnetic field, superparamagnetic nanoparticles do not exhibit magnetization, with their remanence being equal to zero. This behavior results in an S-shaped (sigmoidal) M-H curve, as illustrated in the inset of Figure 13.

Figure 13 presents the magnetization (M–H) curves for bio-EP-MnP/MnP-CA composites containing iron oxide nanoparticles coated with citric acid (CA), illustrating their magnetic response as a function of the applied magnetic field. The sigmoidal shape of the magnetization curves confirms the presence of superparamagnetic nanoparticles within the polymer matrix. It is observed that the saturation magnetization (*M*s) increases proportionally with nanoparticle content, regardless of whether functionalization has occurred. In the bio-EP-MnP composites, the saturation magnetization increases with higher MnP content. A similar trend is observed in the bio-EP composites filled with MnP-CA particles; however, due to better dispersion, the saturation magnetization values are slightly lower compared to those with unmodified MnP nanoparticles. It is well established that saturation magnetization is related to the nanoparticle size and tends to increase with the particle size. The observed trend suggests that nanoparticle agglomeration occurs at higher concentrations in the composites containing bare MnP nanoparticles, likely due to dipole–dipole interactions.

In general, the improved dispersion of MnP-CA nanoparticles in the bio-EP matrix is supported by multiple complementary analyses, including zeta potential, DMA, TGA, and ATR-FTIR. These results collectively indicate that citric acid functionalization enhances nanoparticle compatibility with the epoxy matrix, reducing agglomeration and facilitating stronger filler–matrix interactions. This improved dispersion is a key factor behind the enhanced mechanical, thermal, and curing behaviors observed in EP-MnP-CA composites.

## 4. Conclusions and Discussion

This study highlights the transformative potential of bio-epoxy (bio-EP) nanocomposites incorporating MnPs and their MnP-CA counterparts to revolutionize the field of advanced materials. The integration of MnP and MnP-CA into bio-EP resin systems resulted in a remarkable enhancement of curing efficiency, thermal stability, mechanical properties, and surface functionality, showcasing the unique advantages of nanoparticle-based reinforcements. Bio-EP nanocomposites incorporating both bare and surface-functionalized MnPs were prepared, and their structural, thermal, curing, and surface properties were systematically studied. These nanocomposites present an innovative solution for enhancing the performance of bio-based epoxy resins by leveraging the unique properties of nanoparticles. Incorporating iron-oxide nanoparticles into epoxy resins has been identified as a promising approach to improve curing efficiency, thermal stability, and the mechanical properties of the resulting composites. The nanoparticles were synthesized through precipitation in an aqueous solution and subsequently functionalized with CA, which introduced carboxyl and hydroxyl functional groups, facilitating enhanced interactions with the resin matrix.

Key findings revealed that the functionalization of MnPs with CA significantly improved nanoparticle dispersion within the resin matrix, enabling stronger interfacial interactions through hydrogen bonding. This improvement in dispersion was attributed to the formation of hydrogen bonds between the functionalized nanoparticles (MnP-CA) and the epoxy resin, as confirmed by ATR-FTIR analysis. Hydrogen bonding not only facilitated a more uniform distribution of nanoparticles within the matrix but also established strong intermolecular interactions, resulting in the superior integration of the nanoparticles into the resin system. In contrast, bare MnPs exhibited poor dispersion due to the absence of functional groups capable of interacting effectively with the bio-EP matrix. This not only enhanced nanoparticle integration but also established a more uniform and robust network structure. The improved compatibility of MnP-CA with the bio-EP matrix led to superior thermal stability, delayed thermal decomposition, and a more efficient curing process, as evidenced by DSC results.

The improved dispersion and bonding of MnP-CA within the bio-EP matrix had a notable impact on the thermal properties of the resin composites. The interaction between MnP-CA and the bio-EP matrix contributed to enhanced thermal stability, as evidenced by thermal analysis. Composites containing functionalized nanoparticles exhibited greater resistance to thermal degradation compared to those filled with bare MnPs. This enhancement in thermal stability can be attributed to the improved compatibility and interaction between MnP-CA and the resin matrix, which effectively restricts chain mobility and delays the onset of thermal decomposition.

DSC further revealed that the incorporation of nanoparticles had a catalytic effect on the curing reaction of the bio-EP resin. This effect was more pronounced with MnP-CA, likely due to the presence of hydroxyl groups on the functionalized nanoparticles, which facilitated interactions with the bio-EP reactive groups. These interactions increased the heat released during the curing process, indicating a more efficient crosslinking reaction. The catalytic behavior of MnP-CA not only reduced the activation energy required for curing but also resulted in a more densely crosslinked network, leading to improved mechanical performance in the final composites.

The mechanical properties of the bio-EP nanocomposites were enhanced by the inclusion of MnPs, particularly MnP-CA. Functionalized nanoparticles contributed to higher tensile strength and modulus, demonstrating the benefits of improved dispersion and stronger interfacial interactions. These improvements make the bio-EP nanocomposites suitable for applications requiring materials with robust mechanical performance. Mechanical testing further underscored the benefits of MnP-CA incorporation, with nanocomposites exhibiting enhanced tensile strength and modulus, affirming their suitability for high-performance applications.

In addition to thermal and mechanical enhancements, the incorporation of MnP and MnP-CA influenced the surface properties of the resin composites. Surface analysis indicated that both EP-MnP and EP-MnP-CA exhibited an anionic charge across nearly the entire pH range. This behavior was attributed to the presence of polar groups on the nanoparticles, which contributed to the overall surface charge of the composites due to the group’s distribution after interacting with the bio-EP matrix. In addition, the surface properties of the composites, characterized by an anionic charge across a broad pH range (above pH 4.3–4.7), open new possibilities for functional coatings, adhesives, and other advanced applications where surface interactions are critical. Furthermore, MNPs contribute to the improved thermal conductivity of the epoxy matrix. This attribute is particularly advantageous in applications requiring efficient thermal management, such as electronic devices and heat dissipation systems. Beyond mechanical and thermal enhancements, the inclusion of MnPs or MnP-CA in bio-EP composites imparts magnetic responsiveness—such as superparamagnetic behavior—to the materials. This functional property opens new avenues for applications in electromagnetic interference (EMI) shielding, magnetic sensors, and actuators. Overall, this study demonstrates that the functionalization of nanoparticles with CA not only enhances their dispersion in the resin matrix but also improves the thermal, mechanical, and surface properties of bio-EP composites. These findings highlight the potential of MnP-CA as an effective additive for advancing the performance of bio-based epoxy systems in various industrial applications. By combining environmentally friendly bio-EP resins with multifunctional nanoparticles, this work offers a novel and scalable approach to achieving superior material properties while aligning with the principles of sustainability and reduced environmental impact. The multifunctionality introduced by MNPs/MnP-CA underscores their potential in the development of next-generation, high-performance, and sustainable bio-epoxy nanocomposites for a wide range of industrial sectors.

The findings underscore the potential of MnP-CA as an effective additive for advancing bio-based epoxy systems, paving the way for next-generation green materials with versatile industrial applications.

## Figures and Tables

**Table 1:** MnP and MnP-CA composites based on bio-EP.

An Abbreviation of Material/Filled with Nanoparticles (wt.%)	0.5	3.0
bio-EP		
EP-MnP	EP-MnP-0.5	EP-MnP-3.0
EP-MnP-CA	EP-MnP-CA-0.5	EP-MnP-CA-3.0

**Table 2:** Dimensions of the samples tested.

Samples	Thickness [mm]	Width [mm]
bio-EP		
EP-MnP-0.5	1.85	5.58
EP-MnP-3.0	1.89	5.53
EP-MnP-CA-0.5	1.91	5.46
EP-MnP-CA-3.0	1.89	5.56

**Table 3:** ATR-FTIR characteristic vibration of bio-EP.

Wavenumber cm^−1^	Assignments
3660	N–H Stretching
3347	N–H, O-H Stretching
3050	C–H Stretching
2966	Asymmetrical C–H stretching CH_3_
2928	Asymmetrical C–H stretching CH_3_
2882	Aldehyde C–H stretching
2868	CH_2_ sym. and asym. stretching
1722	C=O stretching
1684	C=O stretching
1608	N–H bending
1580	N–H bending
1511	Deformation vibration mode of CH_3_
1460	C–H deformation, CH_2_
1390	Symmetrical C–H deformation, CH_3_
1293	C–O stretching
1239	C–H in plane bending
1178	C–H in plane bending
1080	C–H in plane bending
1033	C–H in plane bending
918	Epoxide ring
832	C–H out of plane deformation
532	O–H out of plane vibration

**Table 4:** Time in minutes that it takes for neat EP, all MnP and MnP-CA loads EP-composites to reach 99% conversion at three different temperatures.

	99% Conversion at 60.0 °C [min]	99% Conversion at 80.0 °C [min]	99% Conversion at 100.0 °C [min]
EP-neat	389.41	123.27	43.13
EP-MnP-0.5	329.93	92.89	29.96
EP-MnP-3.0	327.24	99.37	34.31
EP-MnP-CA-0.5	247.5	82.05	28.73
EP-MnP-CA-3.0	294.69	86.12	30.63

**Table 5:** Main parameters obtained from DMA analysis.

Bio-Epoxy/Composites	Storage Modulus [MPa] 20 °C	Tg [°C]
EP-MnP-0.5	3081.2	79.4
EP-MnP-3.0	3306.8	72.3
EP-MnP-CA-0.5	3286.8	62.1
EP-MnP-CA-3.0	3345.7	67.1
bio-EP-neat	2713.4	76.8

## Data Availability

The authors declare that the data supporting the findings of this study are available within the paper. Any raw data files are available from the corresponding author upon reasonable request.
